# Influence of Hydrothermal Modification on Adsorptive Performance of Clay Minerals for Malachite Green

**DOI:** 10.3390/molecules29091974

**Published:** 2024-04-25

**Authors:** Enwen Wang, Teng Huang, Qian Wu, Lanchun Huang, Desong Kong, Hai Wang

**Affiliations:** 1 School of Resources and Environmental Engineering, Anshun University, Anshun 561000, China; 2 School of Environment and Resource, Southwest University of Science and Technology, Mianyang 621010, China; 3 Key Laboratory of Waste Treatment and Resource Recycle, Southwest University of Science and Technology, Ministry of Education, Mianyang 621010, China

**Keywords:** hydrothermal modification, adsorption, clay minerals, malachite green

## Abstract

Artificially modified adsorbing materials mainly aim to remedy the disadvantages of natural materials as much as possible. Using clay materials such as rectorite, sodium bentonite and metakaolinite (solid waste material) as base materials, hydrothermally modified and unmodified materials were compared. CM-HT and CM (adsorbing materials) were prepared and used to adsorb and purify wastewater containing malachite green (MG) dye, and the two materials were characterized through methods such as BET, FT-IR, SEM and XRD. Results: (1) The optimal conditions for hydrothermal modification of CM-HT were a temperature of 150 °C, a time of 2 h, and a liquid/solid ratio 1:20. (2) Hydrothermal modification greatly increased the adsorptive effect. The measured maximum adsorption capacity of CM-HT for MG reached 290.45 mg/g (56.92% higher than that of CM). The theoretical maximum capacity was 625.15 mg/g (186.15% higher than that of CM). (3) Because Al-OH and Si-O-Al groups were reserved in unmodified clay mineral adsorbing materials with good adsorbing activity, after hydrothermal modification, the crystal structure of the clay became loosened along the direction of the c axis, and the interlayer space increased to partially exchange interlayer metal cations connected to the bottom oxygen, giving CM-HT higher electronegativity and creating more crystal defects and chemically active adsorbing sites for high-performance adsorption. (4) Chemical adsorption was the primary way by which CM-HT adsorbed cationic dye, while physical adsorption caused by developed pore canal was secondary. The adsorption reaction occurred spontaneously.

## 1. Introduction

Artificially modified/synthetic mineral adsorbing materials mainly aim to alleviate the disadvantages of some natural minerals as much as possible to fully utilize or greatly manifest their advantages in adsorption performance in order to solve environmental problems [1]. Hydrothermal modification is currently one of the most economical and practical methods for the preparation of new materials and compounds. At kettle pressures of 1 MPa–1 GPa and temperatures of 100–1000 °C, water molecules are in a supercritical or subcritical state, which improves the performance of the chemical reactions in an aqueous solution, achieving the objective of modification and, to some extent, substituting for solid-phase reactions at high temperature [2,3]. Since the homogeneous/heterogeneous nucleation mechanism of the hydrothermal reaction differs from the diffusion mechanism of solid-phase reactions, this method creates new features not obtained by other methods [4].

In recent years, Chinese and foreign scholars have applied common hydrothermal methods to prepare environmentally friendly mesoporous/porous functional materials for environmental purification. The raw materials are mainly classified as follows: (1) aluminosilicates, such as rectorite [5,6,7], kaolinite [8,9], bentonite [10,11] and attapulgite [12]; (2) industrial solid waste, such as coal gangue [13], iron ore tailings [14], blast furnace slag [15,16], waste vinasse and red mud [17]; and (3) agricultural solid waste, such as plant leaves [18], peanut shells [19], rice husks and chicken manure [20].

Although purely silicon-based ordered mesoporous materials have been widely applied in numerous fields, they possess numerous disadvantages such as poor thermal stability, few surface activity sites and low ion exchange capacity. In order to solve these problems, scholars have started to utilize aluminum in some natural ore compositions for the preparation of mesoporous silicon aluminum materials by a hydrothermal method. Such materials mainly use clay as their matrix. In 2020, a group successfully loaded TiO_2_ into clinoptilolite through hydrothermal modification to control the crystal phase and particle size of zeolite, with good applications [21]. In 2021, MoS_2_@Kaolin was prepared by a facile one-step hydrothermal method, and the adsorption capacity of it was 1.74 and 16.95 times that of single MoS_2_ and kaolinite, respectively [22]. The mesoporous polymetallic silicate adsorbents prepared with superior adsorption capability towards MB and TC were synthesized by a simple one-pot reaction process, and the specific surface areas of the prepared adsorbent (348.13 m^2^/g) reached 5.9 times that of original clay [23]. In 2022, the zirconium-based pillar support bentonite (Zr-Bent) was prepared through hydrothermal synthesis, whose adsorption capacity of anionic dye Congo red was more than 400 mg/g [24]. Zeolite was first synthesized through simple hydrothermal modification in China, using bentonite as the raw material under the condition of no template agent, and then modified through CaCl_2_ to obtain MSCa, whose maximum adsorption capacity for Pb(II) reached 345 mg/g [25]. *γ*-Fe_2_O_3_ and bentonite were hydrothermally treated to obtain *γ*-Fe_2_O_3_/Mt, which increased the adsorption capacity of *γ*-Fe_2_O_3_ on rhodamine B (RhB) 71.86% over that of bentonite [26]. The calcination and oxidant-assisted hydrothermal treatment was used to synthesize high-performance composite adsorbing materials by regenerating/recycling the abandoned rectorite adsorbent. The respective adsorption capacities for methylene blue (MB) and basic red 14 (BR) were 176.77 mg/g and more than 300 mg/g [27]. Clay was added to brewing residues to obtain DG-A15 following hydrothermal modification, whose respective adsorption capacities of methylene blue and phosphorus were 340.3 mg/g and 97.4 mg/g. In short, hydrothermal modification is a good way to synthesize and modify clay minerals [28].

This study aims to evaluate the adsorption capacity of available clay minerals (CMs), including sodium bentonite (montmorillonite), expanded graphite, rectorite and metakaolin, to remove MG from aqueous solutions. The equilibrium adsorption isotherm is established by the Freundlich and Langmuir models. Kinetic adsorption models are also established in order to analyze the kinetics and mechanisms of dyes adsorption, and thermodynamics are also employed to examine the adsorption results. This study also provides novel insights into preparing materials of adsorption. The modification of adsorbent materials increases the removal capabilities and, in a certain way, is a viable alternative for remediation effects.

## 2. Materials and Methods

### 2.1. Materials

Four raw minerals (sodium bentonite, graphite, metakaolin and rectorite) were obtained from Jianping County in Liaoning Province, Jixi County in Heilongjiang Province, Jinshan Metakaolin Co., Ltd. of Enshi City in Hubei Province, China and Mingliu Rectorite Science and Technology Ltd. in Hubei Province, China. All minerals were crushed to a particle size not more than 74 µm, and then dried in an oven at 105 °C for more than 3 h. Malachite green (MG) and carboxymethyl cellulose sodium (CMC-Na) were provided by Sinopharm Chemical Reagent Co., Ltd. (Shanghai, China). The dye solution was prepared to the required concentration using ultrapure water.

### 2.2. Preparation of Materials

#### 2.2.1. Expanded Graphite

Expanded graphite was obtained by burning intercalated graphite, consisting of graphite, potassium permanganate (KMnO_4_, AR) and perchloric acid (HClO_4_, GR), with respective ratios of 10.0 g, 1.0 g and 10.0 mL at 900 °C for 10–20 s in a muffle furnace. The mixture then underwent reaction for more than 3 h in a round-bottom flask, was washed with ultrapure water to pH 6.0–7.0 and dried below 65 °C.

#### 2.2.2. Hydrothermal Modification of Base Material

The three raw clay minerals (sodium bentonite, rectorite and metakaolinite) were mixed with deionized water according to the compound proportions of minerals in the CM by stirring for 10 min at mass ratios of 5:8:8. Then, the mixture was put into a 100 mL hydrothermal reaction kettle with internal polytetrafluoroethylene coating. After reacting at a certain temperature for a certain time (specific values are shown in Section 3.2.1 and Section 3.2.2), the mixture was taken out, oven dried and ground into particles of size 0.074 μm to obtain hydrothermally modified mixed powder material (tests are shown in Section 3.1).

#### 2.2.3. Granulation of CM-HT

The powder was uniformly mixed with expanded graphite and CMC-Na at the mass ratios of 105:20:18. Then, deionized water was added for granulation (Ø 8 mm). The heating speed was set at 200 °C/h in an oxygen-enriched atmosphere, and the calcination temperature was set at 800 °C. After calcination for 4 h, CM-HT was obtained.

### 2.3. Characterization of Adsorbents

The phase compositions of the CM were analyzed on a rotation anode high power X-ray diffractometer (RU-200B/ D/MAX-RB, Rigaku, Tokyo, Japan) over a 2θ range of 3–70°. The morphological features of the adsorbents were obtained by a scanning electron microscope (JSM-5610LV, JEOL Electronics Co., Ltd., Tokyo, Japan) at an accelerating voltage of 5 kV. Their functional groups were analyzed using Fourier transform infrared spectroscopy (IS-10, Nicolet, Waltham, MA, USA) with a wavenumber range of 400–4000 cm^−1^. Their Brunauer–Emmett–Teller specific surface area was measured by an automatic analyzer of specific surface area and porosity and calculated by the BET method using a P/P0 range from 0.05–0.2.

The test temperature and time for determination of the sample ignition loss were 950–1000 °C and 15–20 min respectively, evaluated using Formula (1):

(1) $$\psi =\frac{m_{0}-m_{1}}{m_{0}}\times 100\%$$

where *Ψ* is the sample ignition loss (%); *m*_0_ is the weight of the uncalcined adsorbents (g); and *m*_1_ is the weight of the calcined adsorbents (g).

### 2.4. Adsorption Test

Adsorption was tested in a rotary shaker (HZQ-C, Harbin Donglian Electronic Technology Development Co., Ltd., Harbin, China) at 110 rpm and 15–45 °C. A quantity of CM or CM-HT was added to 100 mL of the known initial concentration of MG. The time and temperature of hydrothermal modification, liquid/solid ratio, concentration of adsorbent, initial concentration of the dye, initial pH of aqueous solutions and time of adsorptive reaction were investigated in order to evaluate their influence on adsorption potential. At the end of the adsorption equilibration period, the adsorbing material was separated by centrifugation (LXJ-IIB, Anting Scientific Instrument Factory of Shanghai, China) at 5000 rpm for 15 min. The supernatant was then filtered using a Millex VX filter (Millipore 0.45 μm, Burlington, MA, USA) to ensure the solution was free of granules before measuring the residual dye concentration. Finally, the solution was analyzed using a UV-visible spectrophotometer (UV-3000PC, Mapada, Shanghai, China) at *λ*_max_ = 618 nm.

The dye concentration was determined colorimetrically by measuring the maximum adsorption capacity at a wavelength of 618 nm, then a calibration curve was plotted between the adsorption capacity and dye solution concentration. The MG removal ratio was calculated from the calibration curve.

### 2.5. Static Regeneration Test

The method for static regeneration was the same as for the preparation of CM (Section 2.2.2). The subsequent static regeneration material test conditions also were in keeping with the adsorption test (Section 2.4).

### 2.6. Conditions and Variables of the Tests

#### 2.6.1. Temperature

The liquid/solid ratio was set at 1:5, reaction time was 5 h and hydrothermal modification temperature was adjusted between 100 °C, 150 °C, 200 °C and 250 °C. Then, the hydrothermal modification products were mixed for granulation at a certain proportion with the expanded graphite, CMC-Na, etc. (Section 2.2.3). The resulting CM-HT (hydrothermally modified material) was used to adsorb MG cationic dye in order to investigate the influence of hydrothermal modification temperature on the adsorption performance of the CMs.

Conditions for the adsorption test were as follows: concentration of adsorbent was 2.0 g/L; constant temperature was 35 ± 1 °C; vibration frequency of the air bath shaker was 110 rpm; concentration of the MG dye was 500 mg/L; volume of dyed wastewater was 100 mL; reaction container was a 250 mL conical flask; and sampling interval was 10 min.

#### 2.6.2. Time

Based on the first test, the conditions for hydrothermal modification were adjusted as follows: the temperature was set at 150 °C, while the gradient for modification time was set at 0.5 h, 1 h, 2 h, 3 h, 4 h and 5 h.

Test conditions were as follows: concentration of the adsorbent was 2.0 g/L, constant temperature was 35 °C, vibration frequency of air bath shaker was 110 rpm, concentration of MG dye was 500 mg/L, volume of dye wastewater was 100 mL, reaction container was a 250 mL conical flask and sampling interval was 1 h.

#### 2.6.3. Liquid/Solid Ratio

Conditions for the hydrothermal modification test were as follows: the liquid/solid ratio was adjusted to 1:2.5, 1:5, 1:10, 1:15, 1:20 and 1:25; the time was set at 2 h; and other conditions were the same as in Section 3.1.2.

Conditions for the adsorbing dye test were as follows: adsorbent concentration was 2.0 g/L, constant temperature was 35 °C, vibration frequency of air bath shaker was 110 rpm, concentration of MG dye was 500 mg/L, volume of wastewater was 100 mL, reaction container was a 250 mL conical flask and sampling interval was 1 h.

#### 2.6.4. Adsorbent Concentration

(1) Test conditions

A total of 100 mL of MG dye wastewater at a concentration of 500 mg/L was first drawn and then put into a 250 mL conical flask. CM-HT was added by weight, then the flask was put into an air bath shaker. The reaction lasted for 48 h at a temperature of 35 ± 1 °C with a vibration frequency of 110 rpm. The sample was collected for testing.

(2) Test variables

Gradients for the CM-HT concentration were 1.0 g/L, 2.0 g/L, 3.0 g/L, 4.0 g/L and 5.0 g/L.

#### 2.6.5. Initial Dye Concentration

(1) Test conditions

A total of 100 mL of dyed wastewater at a certain concentration was first drawn and then put into a 250 mL conical flask. CM-HT was added at a weight of about 2.0 g/L, then the flask was put into an air bath shaker. The reaction lasted for 48 h at a temperature of 35 ± 1 °C with a vibration frequency of 110 rpm. Then, the sample was collected for testing.

(2) Test variables

Gradients for the initial concentration of MG were 500 mg/L, 750 mg/L, 1000 mg/L, 1250 mg/L and 1500 mg/L.

#### 2.6.6. Adsorption Time

(1) Test conditions

A total of 100 mL MG dye wastewater at a concentration of 1000 mg/L was first drawn and then put into a 250 mL conical flask. CM-HT was added at a weight of about 2.0 g/L. Then, the flask was put into an air bath shaker. The reaction lasted for a certain duration at a temperature of 35 ± 1 °C with a vibration frequency of 110 rpm. The sample was collected for testing.

(2) Test variables

Gradients for the adsorption time were 2 h, 4 h, 6 h, 8 h, 10 h, 12 h, 14 h, 24 h, 26 h and 28 h.

#### 2.6.7. Initial pH

(1) Test conditions

A total of 100 mL of MG dye wastewater at a concentration of 1000 mg/L was first drawn and then put into a 250 mL conical flask. The pH was adjusted in both directions. CM-HT was added at a weight of about 2.0 g/L. Then, the flask was put into an air bath shaker. The reaction lasted for 6 h at a temperature of 35 ± 1 °C with a vibration frequency of 110 rpm. Then, the sample was collected for testing.

(2) Test variables

Gradients for the initial pH (pH_0_) of the dyed wastewater were 0.0, 2.0, 4.0, 6.0, 8.0, 10.0 and 12.0.

#### 2.6.8. Desorbed Recycling of Adsorbents

CM and CM-HT at saturated adsorption were calcined at 750 °C in an oxygen-enriched atmosphere for 1 h. The pH_0_ of the MG cationic dye was adjusted to 6.0. Then, it was recycled five times for comparison.

## 3. Results and Discussion

### 3.1. XRD Quantitative Analysis of Clays

The XRD direct analysis method is also known as the standard curve method. For the content of known phase to be measured, we established a standard curve based on the relative intensity for the same diffraction peak of a standard sample. Using the standard curve calculation, the content of the phase to be measured in the sample could be obtained.

#### 3.1.1. Establishment of the Standard Curve

(1) The standard curve of pyrite

**Figure 1:**
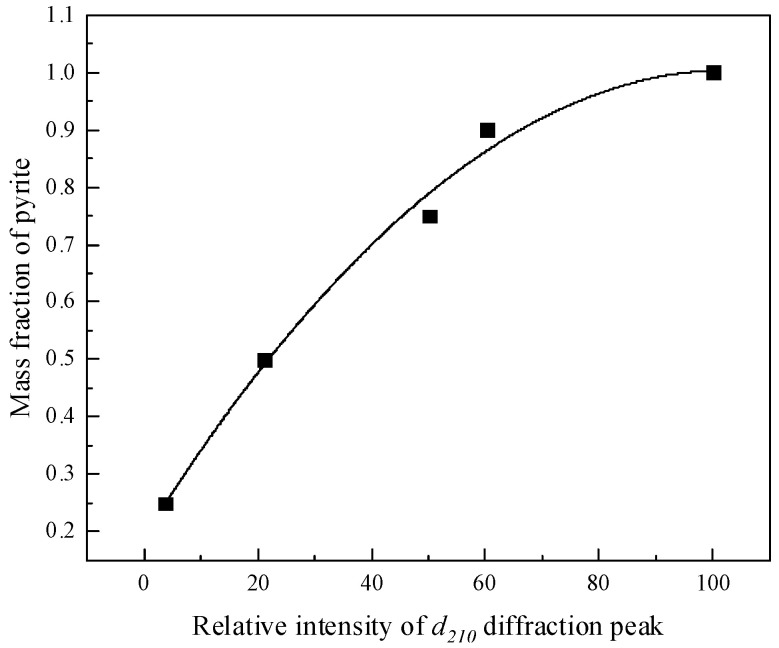
Standard curve of pyrite.

**Figure 2:**
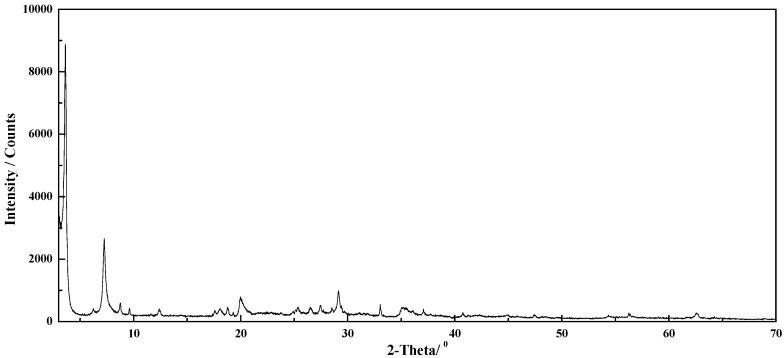
XRD of rectorite.

XRD testing was conducted by a group of standard samples with known pyrite content prepared using the pure pyrite and rectorite in different mass ratios. The integral intensity of the strongest diffraction peak of pyrite (d_210_ = 0.242 nm) in the XRD spectra of each sample was compared with the integral intensity of the same diffraction peak of pure pyrite. A set of data for relative intensity values as X-axis and a pyrite mass fraction as Y-axis were obtained. All the data were subjected to quadratic polynomial regression fitting to obtain the quantitative standard curve of pyrite, as shown in Figure 1, while the XRD of rectorite used in this text is presented in Figure 2.

The regression equation in Figure 1 is calculated as:

(2) $$Y=0.19294+0.01571X-0.00007605X^{2}$$

while R^2^ = 0.984.

(2) The standard curve of quartz

**Figure 3:**
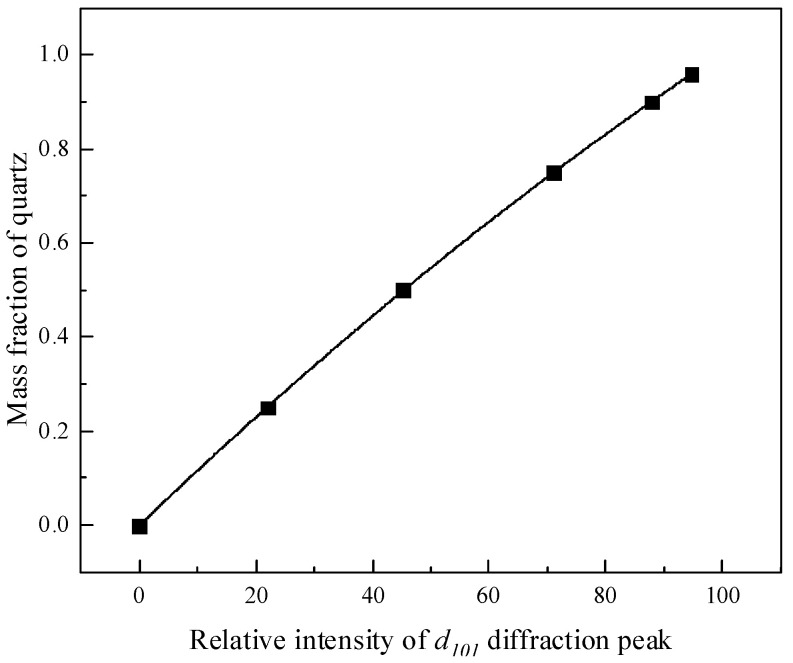
Standard curve of quartz.

**Figure 4:**
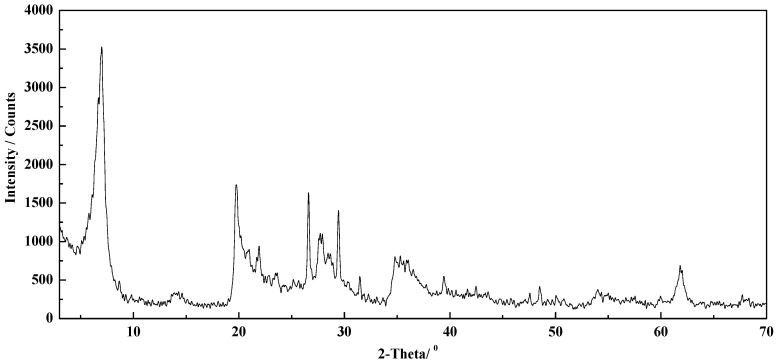
XRD of montmorillonite.

Similarly, XRD testing was conducted by a group of standard samples with known quartz content prepared using the pure quartz and montmorillonite in different mass ratios. The integral intensity of the strongest diffraction peak of quartz (d_101_ = 0.335 nm) in the XRD spectra of each sample was compared with the integral intensity of the same diffraction peak of pure quartz. A set of data for relative intensity values as X-axis and a quartz mass fraction as Y-axis was obtained. All the data were also subjected to quadratic polynomial regression fitting to obtain the quantitative standard curve of quartz, as shown in Figure 3, while the XRD of montmorillonite used in this text is presented in Figure 4.

The regression equation in Figure 3 is calculated as:

(3) $$Y=-0.000173226+0.01185X-0.0000181661X^{2}$$

while R^2^ = 0.9999.

#### 3.1.2. The Content Calculation

(1) The content of rectorite

We selected different samples of pyrite for XRD analysis. The relative intensity of the strongest diffraction peak (d_210_ = 0.242 nm) of pyrite in the sample was measured to be 1.73 under the above standard curve, as shown in Figure 1. Then, it was substituted into Formula (2), and the pyrite content in the sample was calculated to be 21.99%, so the content of rectorite in the text was 78.01%.

(2) The content of montmorillonite

We selected different samples of quartz for XRD analysis as above. The relative intensity of the strongest diffraction peak (d_101_ = 0.335 nm) of quartz in the sample was measured to be 7.95 under the above standard curve, as presented in Figure 3. Then, it was substituted into Formula (3), and the quartz content in the sample was calculated to be 9.29%, so the content of montmorillonite in the text was 90.71%.

### 3.2. Influence of Hydrothermal Modification Process

#### 3.2.1. Temperature

**Figure 5:**
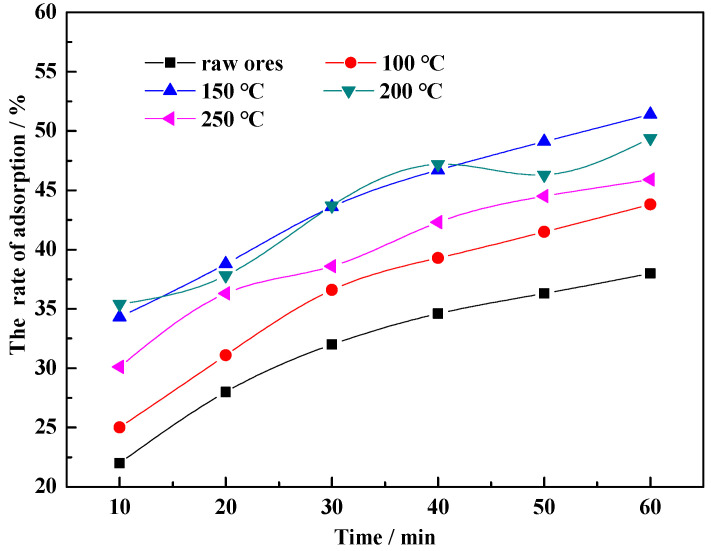
Influence of hydrothermal modification temperature on adsorption efficiency of CM-HT for MG.

Figure 5 shows the influence of hydrothermal modification temperature on the adsorption efficiency of CM-HT. The efficiency of CM-HT was significantly higher than that of unmodified CM, indicating that hydrothermal modification improved the adsorption of MG in purified water. In addition, when the modification temperature was 200 °C, the adsorption effect was unstable; when the temperature was 250 °C, the efficiency decreased significantly. The maximum efficiency occurred in CM-HT obtained at a modification temperature of 150 °C; its adsorption capacity at 1 h reached 128.5 mg/g. The square deviations of the experimental data samples showed significant differences (*p* < 0.05).

Adsorption efficiency first increased and then decreased as the modification temperature increased, which can clearly be attributed to the structural effect of high temperature and pressure on the base material. The relationship between the hydrothermal reaction kettle temperature to saturated vapor pressure is shown in Table 1. As the temperature increased, the saturated vapor pressure increased geometrically [29]. When the temperature was ≤150 °C, the hydrated expansion effect was continuously reinforced with increasing temperature in the system, which increased the interlayer space in the stratified mineral structure [30], thus improving the adsorptive performance. As the temperature rose to >200 °C, however, the base material expanded sharply, causing pore canal collapse at the internal/external surface [31,32] and obstructing adsorbing channels. The number of active adsorbing sites then decreased sharply, directly decreasing adsorption efficiency.

#### 3.2.2. Time

**Figure 6:**
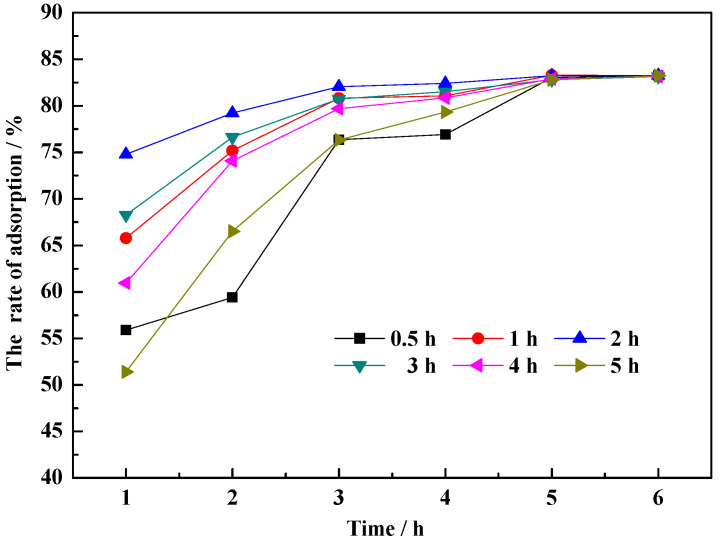
Influence of hydrothermal modification time on adsorption efficiency of CM-HT on MG.

Figure 6 shows the influence of modification time on the adsorption efficiency of CM-HT on MG with a hydrothermal modification temperature of 150 °C. The square deviations of the experimental data samples showed significant differences (*p* < 0.05). At the early stage of adsorption, the efficiency first increased, then decreased with increasing modification time. After 5 h, the efficiency plateaued, indicating that hydrothermal modification time did not influence adsorption capacity but rather resistance by the adsorbents to dye mass transfer. The reasons are given as follows. Since hydrothermal modification increased the interlayer space of the adsorbent, its performance was improved [30]. As the modification time was prolonged from 0.5 h to 2 h, the time to reach equilibrium adsorption capacity was shortened, but, as the time was further prolonged, the excess energy destroyed the stratified crystal structure of the clay, even causing collapse [31,32]. The optimal effect for CM-HT adsorbing purified cationic dye wastewater was achieved when the modification time was 2 h.

#### 3.2.3. Liquid/Solid Ratio

**Figure 7:**
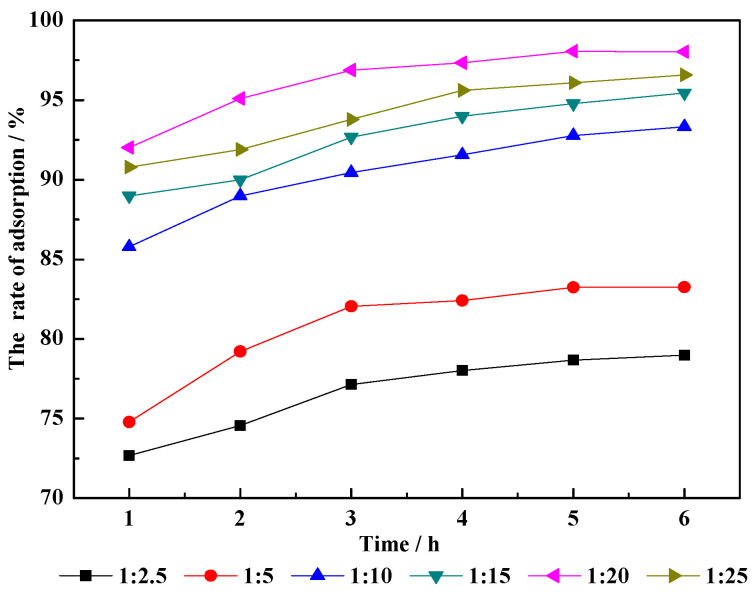
Influence of liquid/solid ratio on adsorption efficiency for CM-HT on MG.

As shown in Figure 7, the liquid/solid ratio significantly influenced the absorption efficiency of CM-HT. The square deviations of the experimental data samples showed significant differences (*p* < 0.05). When the adsorption time was 3 h, adsorption almost reached equilibrium. As the proportion of water to raw minerals in the hydrothermal modification system was increased from 2.5 to 20, the adsorption efficiency increased significantly; when this proportion was further increased to 25, however, the efficiency started to decrease. Therefore, the optimal effect was achieved when the liquid/solid ratio was 1:20.

### 3.3. Characteristics of Adsorbents

#### 3.3.1. Physical Characteristics

Table 2 shows relevant physical parameters of CM-HT. The surface area, pore volume and average micropore/mesopore size data were sourced from BET analysis. CM-HT possesses various advantages over CM, such as more pores, lower scatter ratio and larger specific surface area. Micropore/mesopore analysis showed that the average pore size of both was within 22–27 nm, indicating that CM-HT is a porous material mainly of mesopore distribution.

Table 3 shows the root mean square error of results of physical characteristics of adsorbents. Except for the loss on ignition rate, there was a significant difference in other factors between the two adsorbents.

#### 3.3.2. Change of Pore Size Distribution

**Figure 8:**
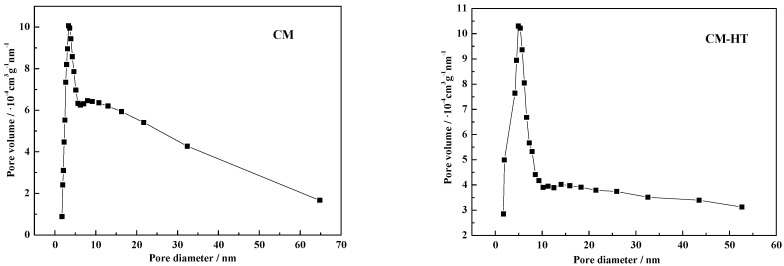
Pore size distribution diagrams for the CM and CM-HT samples.

Figure 8 shows the pore size distribution for CM and CM-HT measured and analyzed through an ASAP 2020M fully automatic specific surface area and porosity analyzer (Micromeritics Instrument Corporation, US). (1) In both samples (CM and CM-HT), a distribution of pore sizes existed. A certain number of pores were found at <2 nm, indicating that the microporous structure distribution in the raw material (CM) was retained in the hydrothermally modified adsorbent (CM-HT) and both materials were good at adsorption of organic cationic dye in wastewater. (2) One sharp narrow peak appeared for both materials near 2–10 nm (more significantly for CM-HT). This peak was wider for CM-HT.

#### 3.3.3. Change of N_2_ adsorption/desorption isotherm

**Figure 9:**
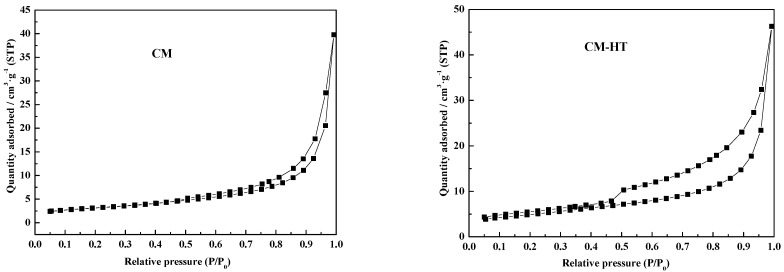
N_2_ adsorption/desorption isotherms for CM and CM-HT samples.

Figure 9 shows the N_2_ adsorption/desorption isotherms for the CM and CM-HT, measured and analyzed through an ASAP 2020M fully automatic specific surface area and porosity analyzer (Micromeritics Instrument Corporation, Norcross, GA, USA). As classified according to IUPAC [33], both isotherms were of Type IV, indicating that both materials had a certain number of mesopores (size 2–50 nm) and macropores (size > 50 nm) [34]. In addition, both samples had Type H4 hysteretic loops in the mid-high pressure area, which was mainly related to slit pores formed by the accumulation of stratified minerals (such as aluminosilicate and expanded graphite). Both hysteretic loops were basically the same at the high-pressure inflection point, but the inflection point of CM-HT was more advanced than that of CM in the low-pressure area (i.e., it moved toward the low-pressure area). Therefore, hydrothermal modification was favorable for mesopore formation, helping improve adsorption performance.

#### 3.3.4. Change of Phase Composition and Crystal Structure

**Figure 10:**
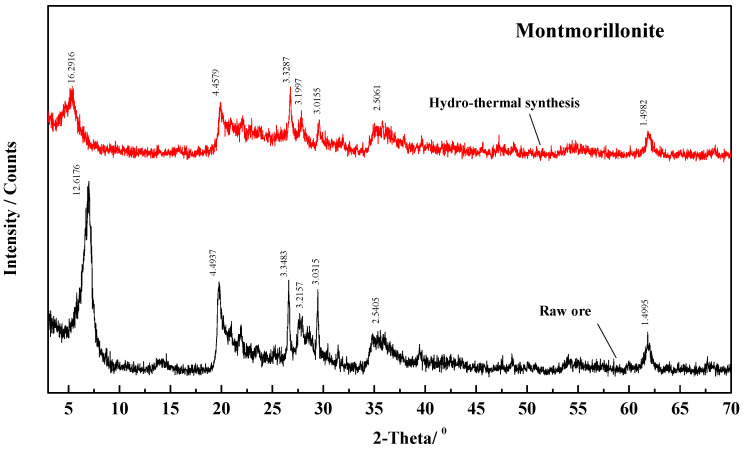

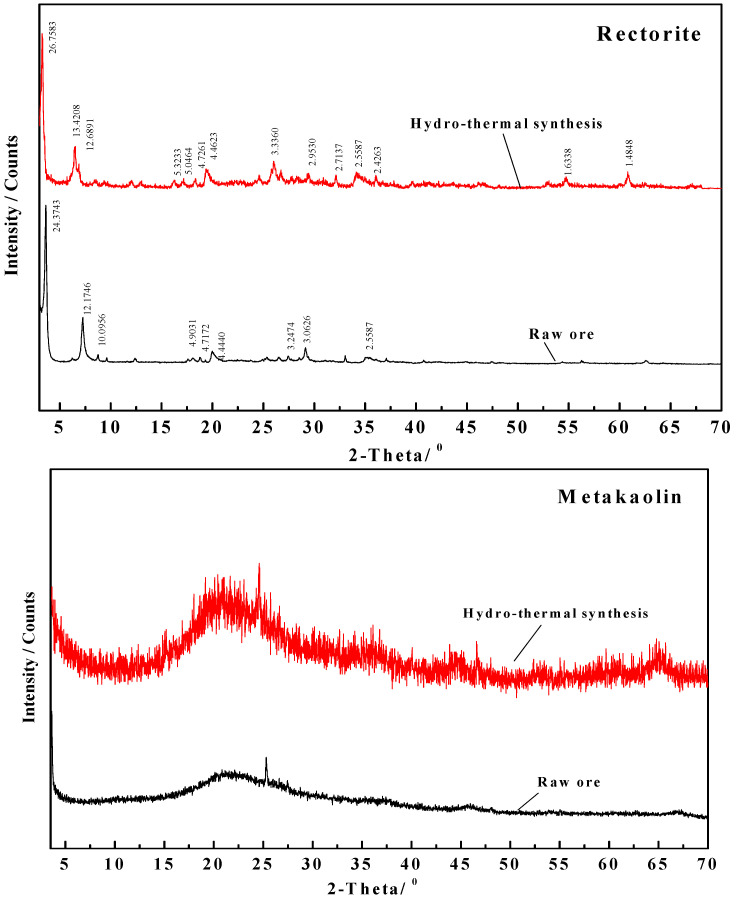
XRD diagram for three raw and hydrothermally modified clay minerals.

Figure 10 is an XRD diagram of crude ore and hydrothermal modification of the three minerals (i.e., sodium bentonite, rectorite and metakaolinite). (1) The interlayer space was increased under certain hydrothermal modification conditions (Table 4). This increase occurred in the 001 direction. The d value was basically unchanged, indicating that the crystal structure of the hydrothermally modified base material became loose only along the c axis direction but was basically unchanged along the b axis. The reason was that hydrated expansion occurred in the base material due to the high pressure of hydrothermal modification [31,32]. (2) After the hydrothermal modification, the background of the base materials in the XRD diagram deepened. The number of burrs increased, and the peak type widened and deflected toward the low-angle area, indicating that the crystal structure of the three base material minerals changed to a certain extent.

(1) Change in interlayer spacing of clay minerals

The above analysis shows that, in the CM-HT prepared under the optimal conditions for hydrothermal modification, the interlayer space of the base material increased along the c axis. As a result, the specific surface area, pore volume, porosity and pore size all increased, which accords with the conclusion in Section 3.3.1.

(2) Change in phase composition and crystal structure

**Figure 11:**
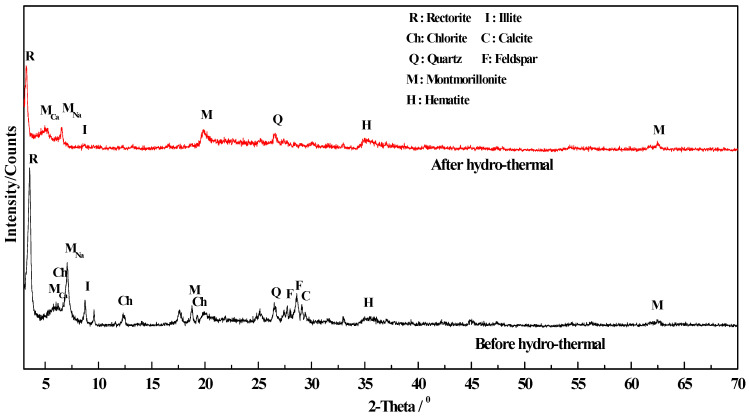
Diagram comparing the precursors of CM-HT before and after hydrothermal modification.

Figure 11 compares XRD analysis before and after hydrothermal modification of the CM-HT precursors (the three base material minerals, i.e., rectorite, metakaolinite and sodium bentonite, mixed at certain ratios). After modification, the concentration of minerals with poorer adsorptive performance (such as feldspar, hematite, chlorite, calcite, illite and quartz) was decreased in the base minerals, thus optimizing and purifying the base material. The decomposition of feldspar under hydrothermal modification is shown in reaction Formula (4) [35].

(4) $$HAlSi_{3}O_{8}+(3n+2)H_{2}O↔3{\left(SiO_{2}\cdot nH_{2}O\right)}^{•}+Al{\left(OH\right)}_{4}^{-}+H^{+}$$

where ${\left(SiO_{2}\cdot nH_{2}O\right)}^{•}$ is the precursor polymer at the silicon-enriched aluminum-unenriched surface; n is the molar number of H_2_O in each mole of precursor polymer; and $HAlSi_{3}O_{8}$ is the hydrated feldspar with conversion into three silicon atoms.

**Figure 12:**
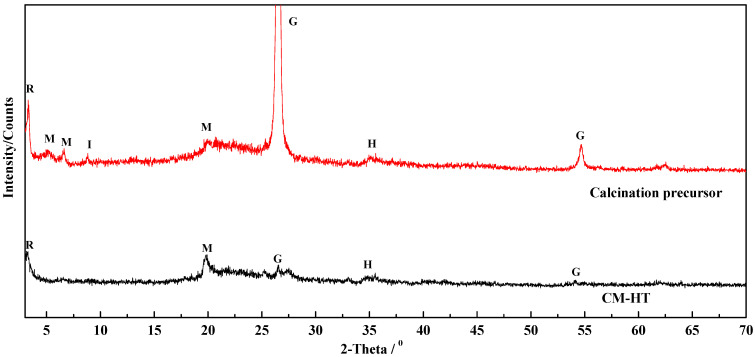
Comparison diagram between CM-HT and its calcination precursor.

Figure 12 compares XRD analysis between CM-HT and the mixed raw minerals before and after calcination. The CM-HT exhibited a characteristic graphite peak, making it a kind of expanded graphite with strong adsorption ability for cationic dye. On the other hand, since the hydrated expansion effect of rectorite/montmorillonite was reinforced following hydrothermal modification, and the interlayer space was increased [30], the *d*_001_ value deflected toward the small angle of 2θ, so interlayer metal ions (such as Na^+^ and Ca^2+^) were exchanged to form holes in the surface and within the interface of the mineral crystal. The resulting macroscopic effect was the formation of pore canals. This was one of the ways by which hydrothermal treatment loosened the crystal structure of the clay and increased the number of crystal defects and highly active adsorbing sites. Further analysis of this mechanism was made through FT-IR analysis in Section 3.3.6.

#### 3.3.5. Analysis of Micrographic Structural Features

**Figure 13:**
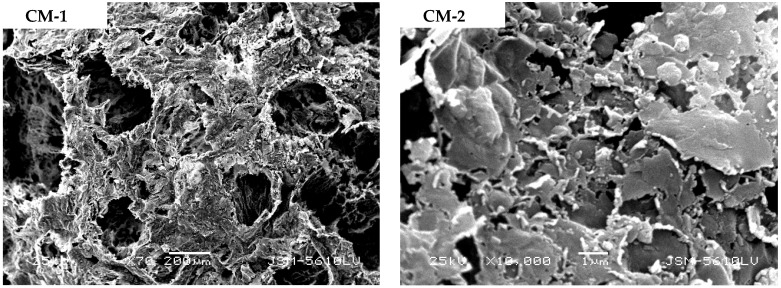

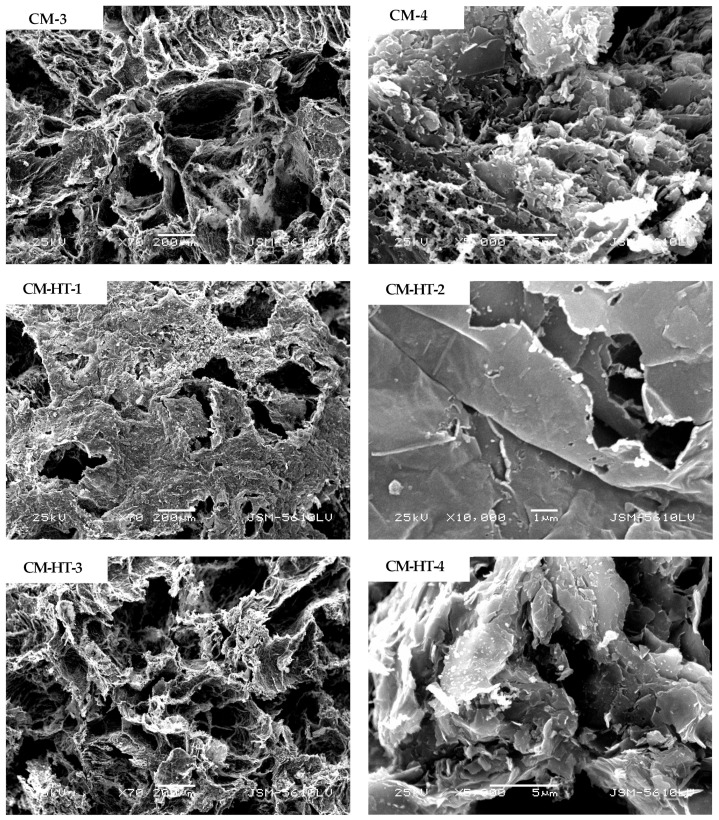
SEM micrograph for the two adsorbents (1 and 2: surface images; 3 and 4: cross-section images).

Figure 13 shows an electron microscopy micrograph of CM and CM-HT following calcination at 800 °C. The surface interfaces of both materials exhibited pore canals and stratification. In the hydrothermally modified CM-HT, the surface was rough, curling was visible, the pore size was relatively uniform, 5–10 μm pores were often found in the cross-section, a honeycombed pore canal network and curled stratified structure were highly developed and a higher degree of development was found in both the internal and external surfaces, which accords with the conclusion of the BET analysis (Section 3.3.1, Section 3.3.2 and Section 3.3.3). The main reasons were given as follows. CM-HT possessed well-developed pore canals formed through high-temperature calcination. Meanwhile, the interlayer space of the clay was increased following hydrothermal modification along the c axis—another main factor for pore formation—laying a good foundation for high adsorption performance [30].

#### 3.3.6. Fourier Transform Infrared (FT-IR) Analysis

**Figure 14:**
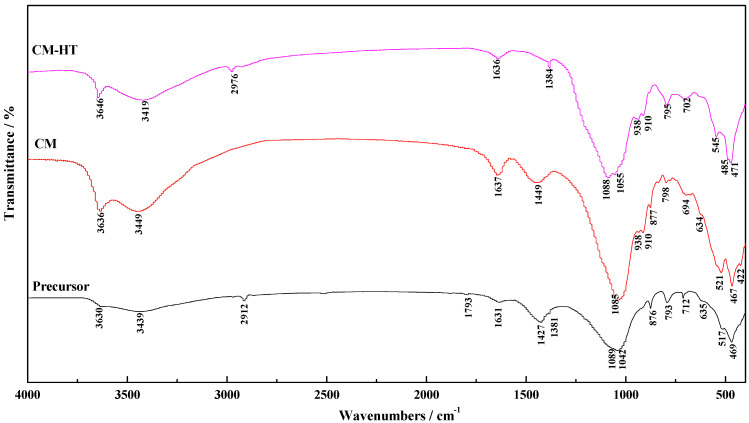
FT-IR spectra of CM, CM-HT and calcination precursor.

Figure 14 compares the FT-IR spectra between the two materials and their calcination precursor. For CM-HT, the equivalent intensities of the two absorption peaks of Si-O bending vibration were opposite to those of the calcination precursor, i.e., the intensity was decreased for the one at the low-frequency side but increased for the one at the high-frequency side [36]. The main reasons were given as follows. After hydrothermal modification, the interlayer space of the montmorillonite in the CM-HT increased [30], and the interlayer metal cations connected with the bottom oxygen were partially exchanged, so the electron cloud of the bottom oxygen deflected toward Si in the silicon–oxygen tetrahedron, and the chemical bonds were strengthened (i.e., the intensity decreased for the absorption peak at the low-frequency side). Moreover, since the electron cloud of the top oxygen deflected toward Si in the silicon–oxygen tetrahedron, the binding force of Si was attenuated for the electron cloud of the top oxygen, and chemical bonds were attenuated (i.e., the intensity increased for the absorption peak at the high-frequency side). In other words, following hydrothermal modification, interlayer metal cations in the montmorillonite phase of the adsorbing material were partially exchanged, so the CM-HT became more electronegative, providing a new path for preparation of materials with strong adsorption performance for cationic dye.

### 3.4. CM-HT Adsorbing Malachite Green

This section aims to investigate the influence of hydrothermal modification on the performance of adsorbents for cationic dye.

#### 3.4.1. Adsorbent Concentration

**Figure 15:**
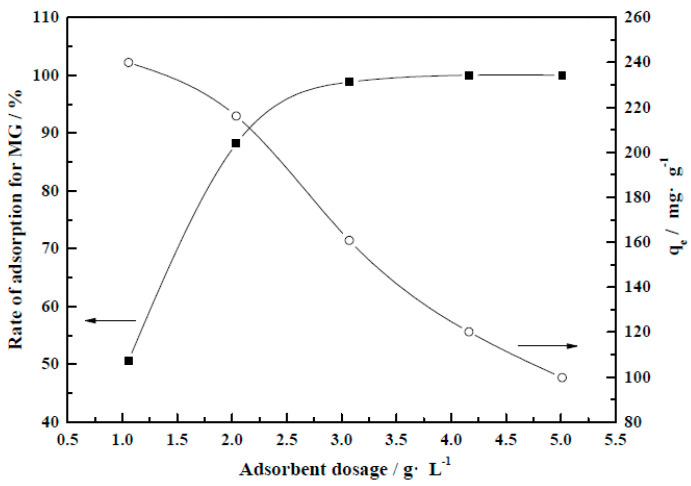
Influence of CM-HT concentration on MG removal rate and equilibrium adsorption capacity (*q*_e_).

As shown by the adsorbent concentration–efficiency curve in Figure 15, the MG removal rate tended to increase with increasing CM-HT concentration, while the equilibrium adsorption capacity (*q*_e_) decreased.

The boundary point was 2.0 g/L. The increase in MG removal rate as the weight of adsorbent was increased within the range of 1.0–2.0 g/L was significantly higher than in the 2.0–5.0 g/L range.

#### 3.4.2. Initial Dye Concentration

**Figure 16:**
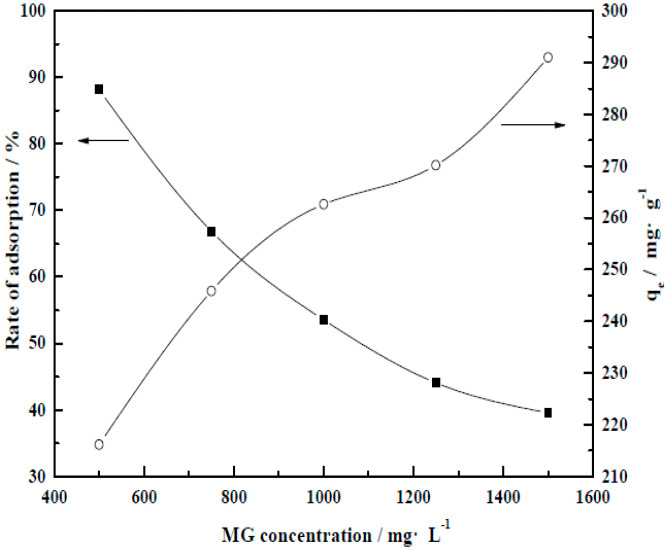
Influence of MG initial concentration on removal rate by CM-HT and equilibrium adsorption capacity (*q_e_*).

As shown in Figure 16, under the premise of a single-factor change in the initial concentration of MG dye, with increasing initial concentration, adsorption efficiency tended to decrease but the equilibrium adsorption capacity (*q_e_*) increased.

As shown by the MG concentration–adsorption rate curve, under the test conditions, the boundary point of dye concentration for adsorption by CM-HT was 1000 mg/L. Therefore, the subsequent experiments used a concentration of 1000 mg/L.

#### 3.4.3. Adsorption Time

**Figure 17:**
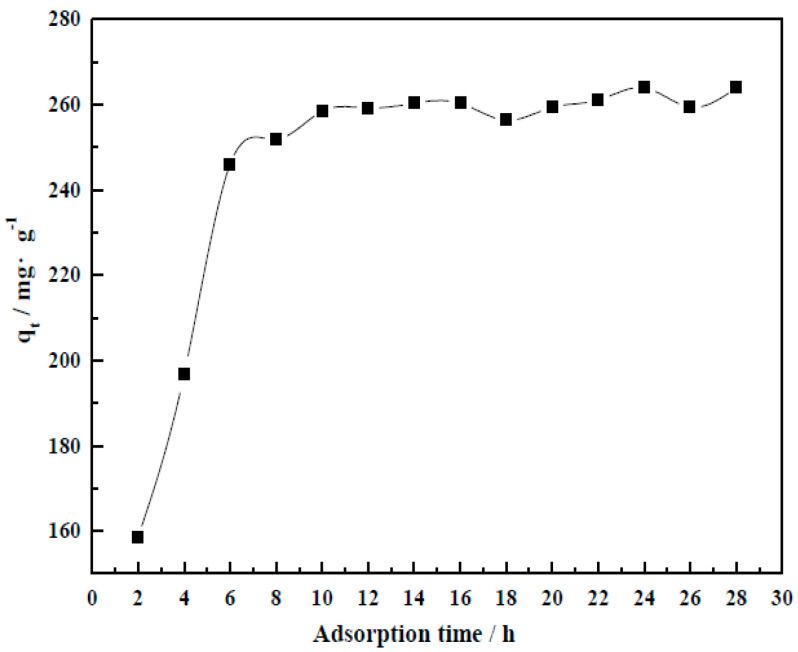
Influence of adsorption time on adsorption capacity (*q_t_*) by CM-HT.

As shown by Figure 17, the adsorption could be divided into two stages of rapid then slow adsorption. After 6 h, adsorption basically achieved an equilibrium. The adsorption capacity was 246.05 mg/g.

#### 3.4.4. Initial pH

According to the literature [37,38], the pH of wastewater is one of the main factors influencing adsorption capacity. This section mainly studies the adsorption/purification effect of CM-HT prepared under optimal hydrothermal modification conditions for wastewater at different initial pH values (pH_0_).

**Figure 18:**
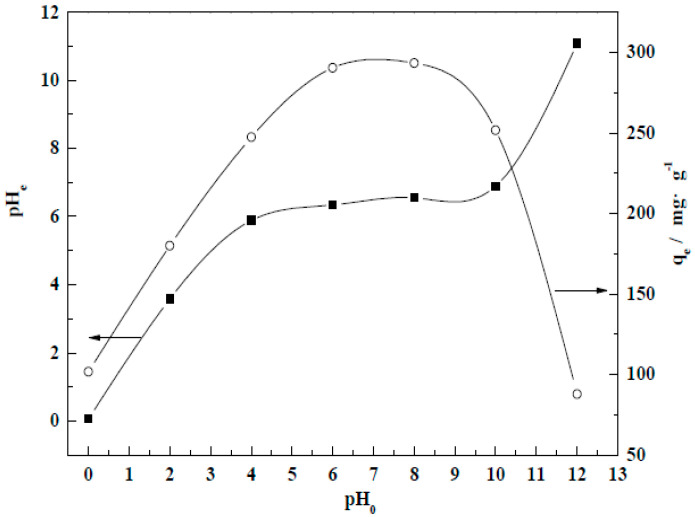
Influence of pH_0_ on equilibrium CM-HT adsorption capacity (*q_e_*) and equilibrium pH (pH_e_).

As shown by Figure 18, the adsorbing/purifying effect of CM-HT on MG dye in wastewater differed significantly by initial pH value (pH_0_) in the absorption system. As pH_0_ gradually increased from 0.0 to 12.0, the equilibrium adsorption capacity (*q_e_*) first increased, then decreased. The maximum *q_e_* (*q_e,max_*) was achieved within the pH_0_ range of 6.0~8.0 (i.e., about 290 mg/g), and was 61% higher than that of CM. H^+^ or Na^+^ in the solution competed with MG for adsorption when pH_0_ was too low or too high [39]. Therefore, it was more conducive to the adsorption of granules when pH_0_ was in the neutral range (the pH of adsorption reaching equilibrium (pH_e_) was basically in a near neutral state). Since the pH_0_ of 1000 mg/L MG solution was about 3.34, considering the environment, the optimal pH_0_ of the solution was 6.0.

#### 3.4.5. Desorbed Recycling of Adsorbents

**Figure 19:**
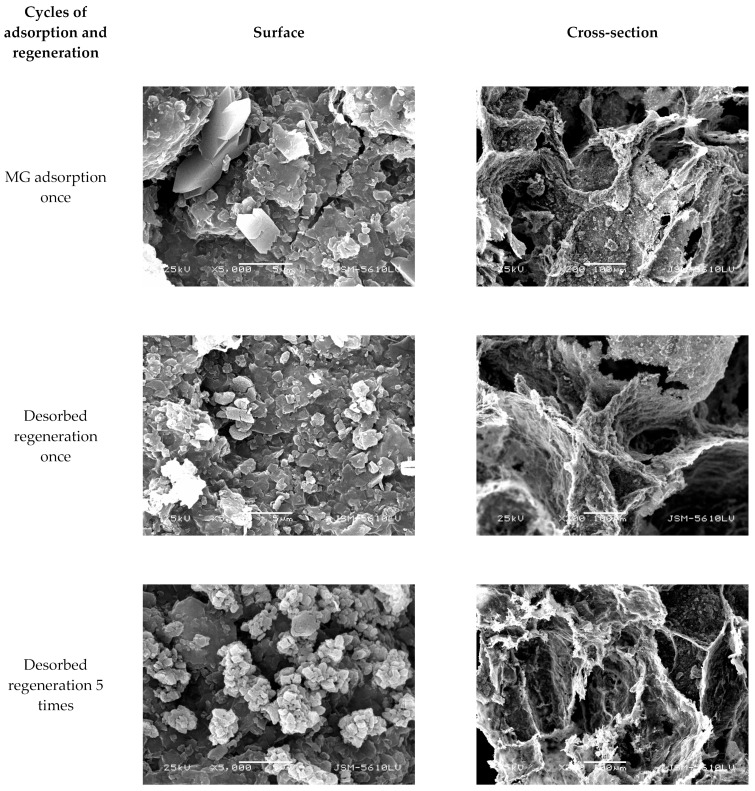
Surface and cross-section SEM micrographs of CM-HT following adsorption and desorption.

**Figure 20:**
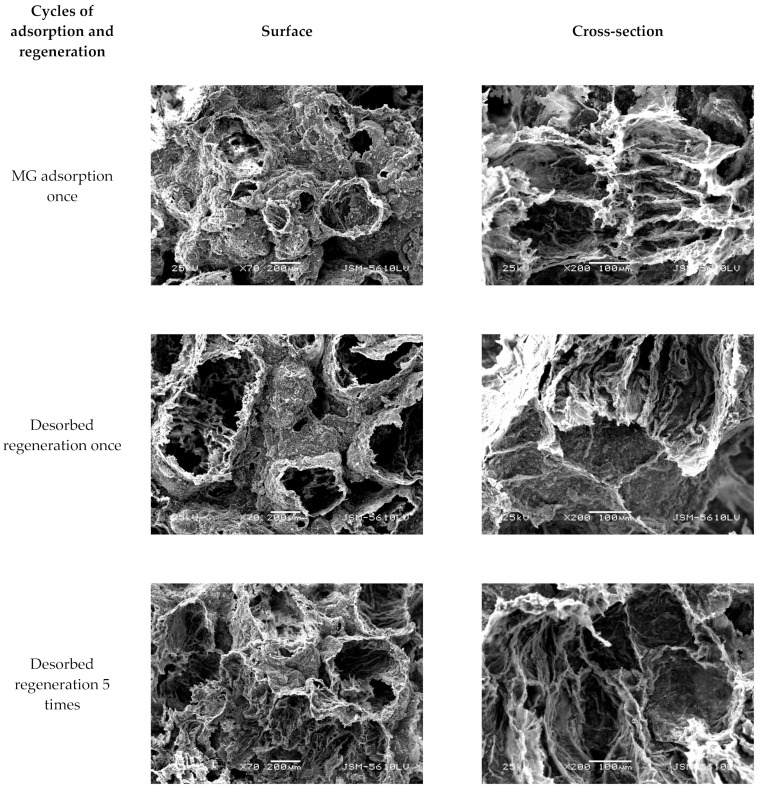
SEM micrographs of surface and cross-section of CM following adsorption and desorption.

**Figure 21:**
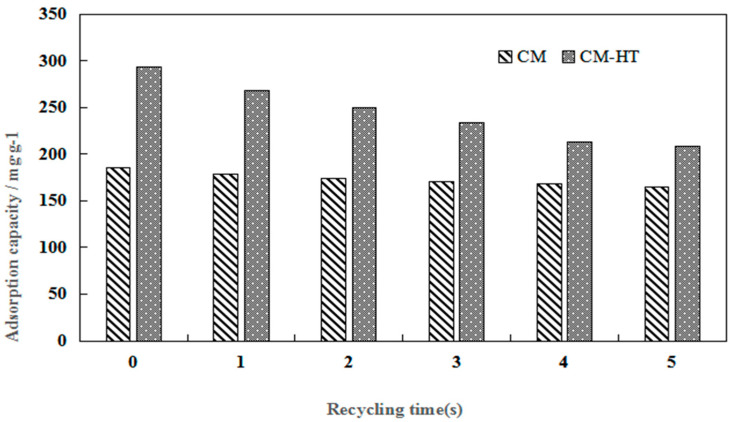
Influence of recycling cycles on adsorptive capacity of CM-HT.

**Figure 22:**
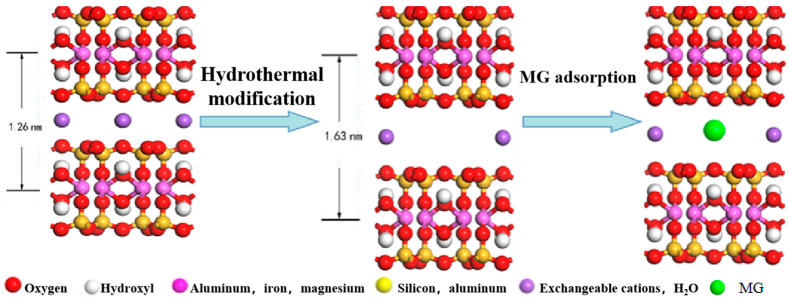
Schematic diagram for hydrothermal modification of montmorillonite and interlayer adsorption.

Figure 19 and Figure 20 show SEM images demonstrating the change in external surface and cross-section of CM-HT and CM, respectively, before and after MG absorption. After the first desorbed regeneration, the flaky structure at the external surface of the CM-HT was damaged more seriously than of the CM, but the pore canals and thickness of pore wall were more developed in the CM-HT than in the CM. As shown by Figure 21, after one generation of recycling, the *q_e,max_* of the CM-HT reached 267.89 mg/g, but the CM was only 178.92 mg/g (Figure 22). Although the flaky structure at the external surface of the CM-HT was damaged more seriously following multiple regenerations, good adsorptive performance was still maintained. After five desorbed regeneration/recycling cycles, the *q_e,max_* of the CM-HT was still 207.97 mg/g. Therefore, CM-HT possesses excellent regeneration and recycling performance.

### 3.5. Effect of Hydrothermal Modification on Adsorptive Performance of Clay-Based Minerals

#### 3.5.1. Mechanism of CM-HT Adsorption

In this section, products before and after hydrothermal modification were used for micrographic feature analysis. SEM analysis was conducted before and after adsorption and after desorption, and FT-IR analysis was conducted before and after adsorption. This section aims to explore the mechanisms for hydrothermal modification in order to improve the adsorptive performance of clay minerals on cationic dye.

**Figure 23:**
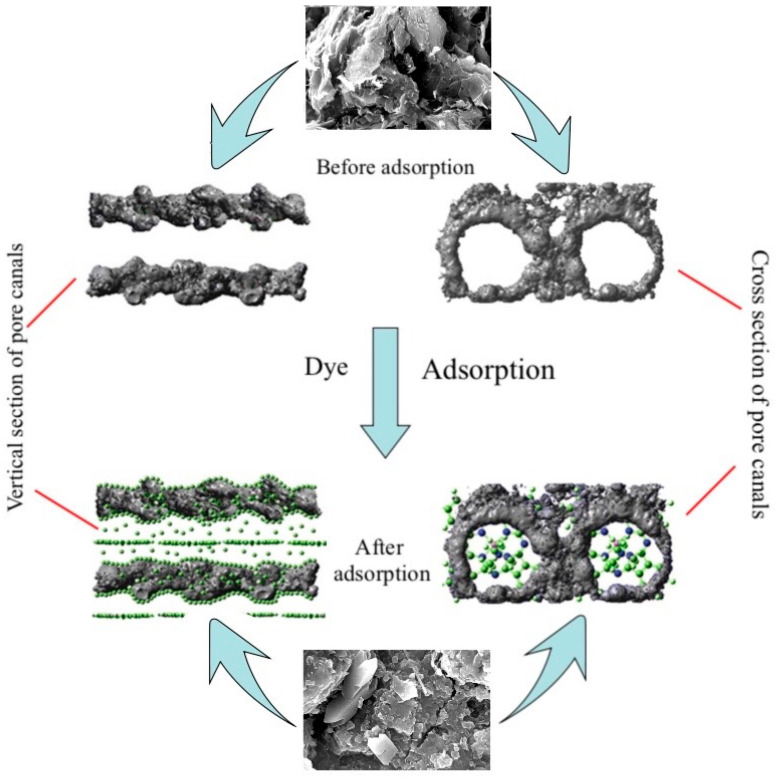
Schematic diagram for CM-HT adsorbing cationic dye.

As shown in Figure 22 and Figure 23, after hydrothermal modification of clay minerals such as montmorillonite, illite and rectorite, the crystal structure became loose along the c axis [30]. The interlayer space was increased. For example, the *d*_001_ value of montmorillonite increased from 12.6176 to 16.2916, *d*_001_ of rectorite increased from 24.0870 to 26.7583 and *d*_002_ increased from 12.1747 to 13.4208 (Table 4). Therefore, the specific surface area of CM-HT was 67.32% larger than that of unmodified CM.

**Figure 24:**
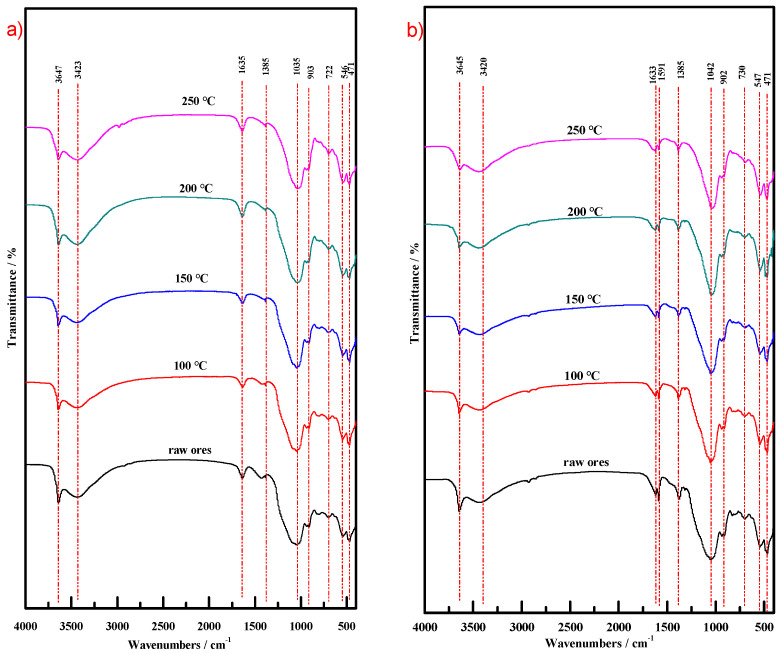
FT-IR spectra of CM-HT (**a**): before MG adsorption; (**b**): after MG adsorption.

Hydrothermal modification increased the interlayer space of montmorillonite in the clay, partially exchanging the interlayer metal cations connected with the bottom oxygen, thus CM-HT became electronegative. Vacancies formed after the exchange of metal cations became active cation adsorption sites. Figure 24 shows the following results. ① Near wavenumber 1591 cm^−1^, the peak intensity and peak type changed somewhat, which was caused by characteristic vibration of aromatic rings; ② Near wavenumber 1385 cm^−1^, the peak intensity and peak type changed somewhat, which was caused by expanding/contracting vibration of Ar-N bonds; ③ Near wavenumber 1170 cm^−1^, the peak type changed somewhat, which was caused by expanding/contracting vibration in the Ar-C bonds. All peaks occurring under the above conditions were characteristic adsorption peaks corresponding to MG, indicating that CM-HT produced an adsorption effect on MG. In the FT-IR spectra following CM-HT adsorption, the peak type and position were changed for both the absorption peak of Si-O expanding/contracting vibration near wavenumber 1035 cm^−1^ and the absorption peak of Al-OH-Al bending vibration near wavenumber 903 cm^−1^. Therefore, CM-HT produced a chemical adsorption effect on MG, which occurred both at the interlayer and at the surface of silicon–oxygen tetrahedral montmorillonite and rectorite.

Chemical adsorption was the primary way by which CM-HT adsorbed cationic dye, while physical adsorption caused by pore canals was secondary. The adsorption reaction occurred spontaneously. Following hydrothermal modification, the specific surface area of CM-HT was increased by 67.32%, and the *q_e,max_* increased nearly 60% to 290.45 mg/g.

#### 3.5.2. Isotherm Analysis

In order to study the surface interaction between the adsorbing material and cationic dye, quantitative analysis and predication were performed using the common adsorption isotherm model of Langmuir and Freundlich.

(1) Langmuir adsorption isotherm model

The Langmuir adsorption isotherm model [40] starts with surface chemicals and selective adsorption at the gas–solid interface. Under the premise of unchanged ambient temperature, when the dye molecules form a single layer saturating the CM or CM-HT (one adsorbing site can only be occupied by one dye molecule), the adsorbent achieves its maximum capacity (*q*_max_) and adsorption and desorption are at thermodynamic dynamic equilibrium. The Langmuir adsorption isotherm equation is expressed by Formula (5):

(5) $$q_{e}=\frac{q_{\max}K_{L}C_{e}}{1+K_{L}C_{e}}$$

where *q_e_* is the equilibrium adsorption capacity per unit of adsorbent (mg/g); *C_e_* is the concentration of adsorbate in solution when the adsorption reaction reaches equilibrium (mg/L); *q*_max_ is the maximum adsorption capacity of a single layer at the adsorbent surface (mg/g); and *K_L_* is the Langmuir constant (L/mg).

Under given conditions, the shape of the Langmuir isotherm model depends on the preset adsorption system. A favorable system for Langmuir adsorption isotherms can be described through a dimensionless factor or equilibrium constant (*R_L_*) [41], as shown in Formula (6).

(6) $$R_{L}=\frac{1}{1+K_{L}C_{i}}$$

where *C_i_* is the initial concentration of dye (mg/L). Possible *R_L_* values are classified into four types in relation to the adsorption isotherm curve. When *R_L_* = 0, an irreversible reaction is considered; when *R_L_* = 1, a linear equation is considered; when *R_L_* > 1, an unfavorable reaction is considered; and when *R_L_* = 0~1, a favorable reaction is considered.

(2) Freundlich adsorption isotherm model

In contrast to the Langmuir adsorption isotherm model [42], the Freundlich adsorption isotherm model describes a complex interface system and reversible adsorption system, not limited to a single-layer interface reaction. Its equation is shown in Formula (7)

(7) $$q_{e}=K_{F}C_{e}^{\frac{1}{n}}$$

where *K_F_* and *n* are constants of the Freundlich adsorption isotherm. *K_F_* represents the adsorption capacity, determined by various factors such as adsorbate/adsorbent characteristics, ambient temperature and adsorbent concentration (the larger *K_F_*, the larger the capacity); a dimensional constant with a unit of mg^1−1/*n*^ L^1/*n*^ g^−1^. *n* represents the linear deviation of the adsorption model; it is related to the nature of the liquid/solid adsorbing system; a dimensionless constant, usually >1. As indicated by the relevant literature, 1/*n* determines the intensity and energy of the adsorption reaction. When 1/*n* = 0.1–0.5, adsorption occurs spontaneously; when 1/*n* > 2, the adsorption reaction is difficult.

**Figure 25:**
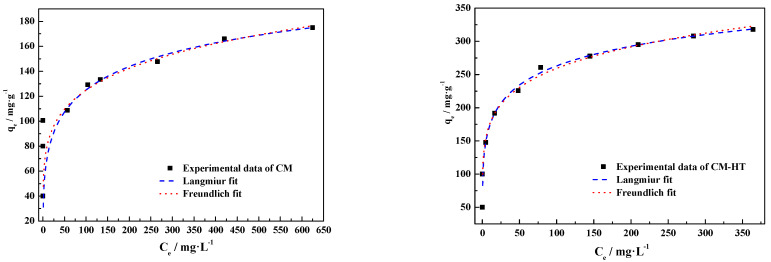
MG adsorption isotherms for both adsorbents (35 ± 1 °C).

As shown by Figure 25, the data of CM and CM-HT adsorbing MG fit well with the Langmuir isothermal adsorption model. The degree of fit with the Freundlich model was lower, indicating that both substances produced a strong chemical adsorption effect [43], which accords with the analytical conclusion in Section 3.5.1.

In addition, as shown by the Langmuir adsorption isotherm model curve-fitting data for CM and CM-HT, the maximum respective fitted adsorption capacities (*q_max,fitted_*) were 310.12 mg/g and 625.15 mg/g, indicating that CM-HT had higher adsorption capacity for MG than CM, which accords with the maximum experimental adsorption capacities (*q_max,exp_*) (Table 5). In the Freundlich adsorption isotherm model, *K_F_* represents adsorption capacity. By analyzing the fit parameter data, the same conclusion was made as the Langmuir model, i.e., the *K_F_* of CM-HT was larger than for CM. In addition, as shown by Table 4, 1/n was within the range of 0.1–0.5 in the Freundlich adsorption isotherm model, indicating that adsorption of cationic dye occurred spontaneously in both materials.

**Figure 26:**
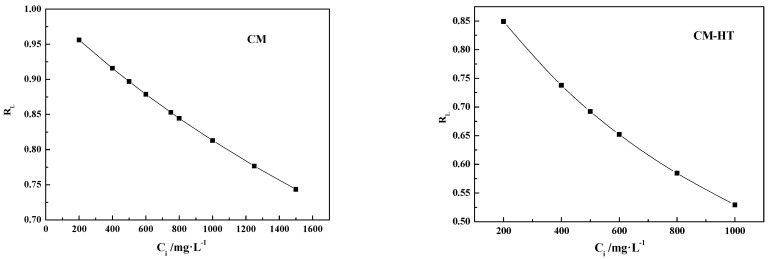
Relation curve for *R_L_* of both adsorbents vs. initial concentration of MG.

Figure 26 is the relation curve for *R_L_* value and initial malachite green concentration of both adsorbents in the Langmuir adsorption isotherm model. The *R_L_* of both CM and CM-HT was within the range of 0.5–0.99, indicating that the test conditions were favorable for smooth occurrence of the adsorption reaction, which accords with the conclusion of the Freundlich model.

#### 3.5.3. Kinetic Analysis

In order to study how the static adsorption process regulates the kinetic adsorption mechanisms and behavior of wastewater dye in hydrothermally modified clay minerals, this section discusses the kinetic performance of CM and CM-HT adsorbing MG (a typical cationic dye). Two kinetic models, a pseudo-first and pseudo-secondary kinetic model, were adopted for analysis and comparison. The time-varying conditions for the adsorption behavior of the two materials for MG in wastewater were analyzed quantitatively, helping reveal the relation between absorbent structure and performance. The two models predicted the adsorption process and results.

(1) Pseudo-first kinetic model

A pseudo-first kinetic equation [44] is proposed and established on the basis of the following hypothesis: Time is the factor influencing change in the adsorbate. When the adsorbate is a solute, this change is influenced by the concentration in the saturated solution. The equation for this model is shown in Formula (8).

(8) $$q_{t}=q_{e}(1-e^{-k_{1}t})$$

where *q_t_* is the mass of dye in the wastewater adsorbed per unit of absorbent at time *t* (mg/g); *t* is the reaction duration (min); and *k*_1_ is the model constant (/min).

The formula for the pseudo-first kinetic model is specified as follows. Under the premise that the ambient factors of the system are known, after the equilibrium adsorption capacity (*q_e_*) per unit of adsorbent is determined, it can be judged whether the unit adsorption capacity (*q_t_*) and corresponding adsorption time conform to the theoretical model. However, since the system is very slow to reach the adsorption equilibrium, *q_e_* is difficult to measure accurately. Therefore, in actual practice, test data for *q_t_* and corresponding adsorption time are often used for linear or non-linear fitting. The degree of fit is then analyzed in order to judge whether it conforms to the pseudo-first kinetic model, and the theoretical equilibrium adsorption capacity (*q_e_*_1_) is calculated under specific conditions.

(2) Pseudo-secondary kinetic model

The equation for pseudo-secondary kinetic model [45] is shown in Formula (9)

(9) $$q_{t}=\frac{k_{2}q_{e}^{2}t}{1+k_{2}q_{e}t}$$

where *k*_2_ is the kinetic adsorption velocity constant for the model (g/mg/min).

**Figure 27:**
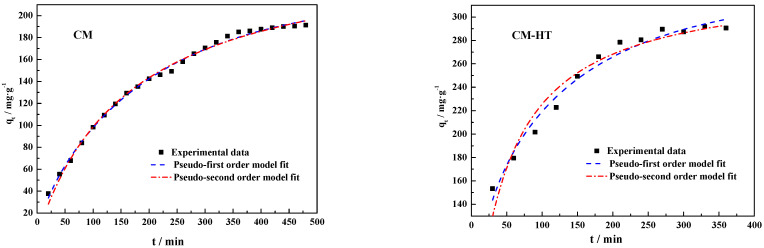
Fits of kinetic models describing CM and CM-HT adsorbing MG at 35 ± 1 °C.

Both kinetic models systematically explore the time-varying conditions of CM-HT adsorbing MG. The model fit curves are shown in Figure 27. Relevant parameters of the models are shown in Table 6.

As shown in Table 6, the non-linear degree of fit was better in the pseudo-first model, with a relation coefficient (R^2^) > 0.97. The theoretical equilibrium adsorption capacity (*q_e_*_1_) from the pseudo-first model better approximated the test data, further indicating that this model better described the time-varying behavior of CM-HT adsorbing cationic dye.

As shown by the overall analysis, the theoretical equilibrium adsorption capacity (*q_e_*_1_) values given by the pseudo-first kinetic model fit the test data (*q_e,exp_*), therefore this model better described the time-varying behavior of CM and CM-HT adsorbing MG in wastewater.

#### 3.5.4. Thermodynamic Analysis

Adsorption is always accompanied by exothermic or endothermic energy changes, as well as other reactions. The magnitude and changes in heat reflect the intensity and changes of the adsorption force (or adsorption bond). Therefore, it reflects the overall change of energy over the whole process [46].

Since adsorption of MG by CM and CM-HT conformed to the Langmuir adsorption isotherm model, this section adopted the Gibbs free energy change model.

By analyzing absorption data at different temperatures (288 K, 298 K, 308 K and 318 K), using Formulas (10) and (11), the following three thermodynamic parameters were calculated: change in Gibbs free energy (Δ*G*), change in entropy (Δ*S*) and change in enthalpy (Δ*H*).

(10) $$\Delta G=-RT\ln(10^{6}K_{L})$$

(11) ΔG=ΔH−TΔS

where *R* is the gas constant (8.314 J/mol/K); *T* is the temperature (K); and *K_L_* is the Langmuir constant (L/mg). Under the conditions of no temperature change or pressure–volume work, adsorption processes were classified as follows: when Δ*G* < 0, adsorption can occur spontaneously; when Δ*G* = 0, the adsorption system is at equilibrium; and when Δ*G >* 0, adsorption cannot occur spontaneously.

**Figure 28:**
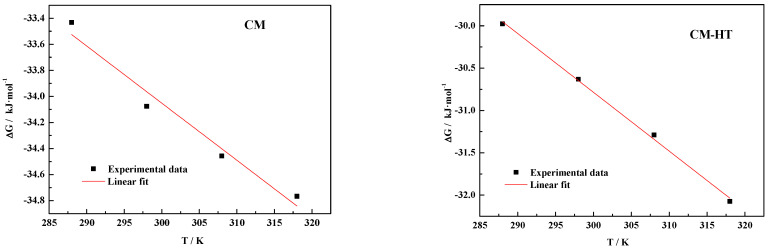
Relation between Δ*G* and T for CM and CM-HT adsorbing MG dye.

Thermodynamic analysis of CM and CM-HT adsorbing MG is shown in Figure 28 and Table 7. At temperatures from 15 to 45 °C, the thermodynamic parameters were similar between CM and CM-HT, i.e., Δ*G* < 0, Δ*H* < 0 and Δ*S* > 0, indicating that both materials spontaneously adsorbed MG.

In addition, Δ*H* < 0 for both materials, indicating that adsorption emitted heat. As shown by further comparison, absolute value of Δ*H* in CM-HT was significantly lower than in CM, indicating that, due to the action of the expanded graphite, unit heat emission was decreased in CM-HT. However, according to the relevant literature [47], the adsorption of cationic dye by expanded graphite is an exothermic process. Therefore, unit heat emission during adsorption by CM can be reduced after combined use with expanded graphite, effectively increasing physical adsorption in the system.

Δ*S* > 0 for both materials, indicating the increased degrees of freedom for the solid–liquid interface indicated that adsorption capacity was improved through hydrothermal modification and by controlling the calcination environment (anoxic calcination).

## 4. Conclusions

In this study, a compound of three clay minerals (sodium bentonite, rectorite and metakaolinite) was hydrothermally modified. CM-HT (a CM) was prepared in an oxygen-enriched atmosphere, and the influence of temperature, time and liquid/solid ratio was explored on the adsorbent micrographic structure, surface functional groups and interface crystal defects at the mineral surface. The following conclusions were made:

1. Temperature of hydrothermal modification. At around 150 °C, phase transformation in the clay (which reduces the concentration of minerals with poorer adsorptive performance in the base material, such as feldspar, hematite, chlorite, calcite, illite and quartz) and hydrated expansion is promoted, increasing the adsorption capacity. As the modification temperature continues to rise, due to overpressure in the reaction kettle, excessive hydrated expansion occurs in the base material, causing the collapse of pore canals at the internal/external mineral surface, obstructing adsorbing channels, reducing the number of active adsorbing sites and decreasing adsorption efficiency.
2. Duration of hydrothermal modification. As the modification time is increased from 0.5 h to 2 h, the interlayer space of the clay is increased due to the hydrated expansion, improving the adsorptive performance of CM-HT and shortening the time to reach equilibrium adsorption. With continued increase in modification time, the saturated vapor pressure is increased, making the mineral crystal structure looser, and even causing collapse, which is unfavorable for MG adsorption.
3. Liquid/solid ratio. As the specific gravity of water in the hydrothermal modification system is increased, interlayer metal cations in the clay are exchanged in CM-HT, creating more active adsorbing sites. When the ratio between water and raw minerals is >20:1, since excessive metal cations are exchanged, collapse is caused in the mineral crystal structure, which is unfavorable for MG adsorption.

In summary, on the basis that Al-OH and Si-O-Al groups are reserved in unmodified CM with good adsorbing activity, hydrothermal modification loosens the crystal structure of the clay along the direction of the c axis, and interlayer space is increased to partially exchange the interlayer metal cations connected with the bottom oxygen, giving CM-HT higher electronegativity, as well as more crystal defects and chemically active adsorbing sites for high-performance adsorption. Therefore, isothermal adsorption is more in line with the Langmuir model. Chemical adsorption is the primary way by which CM-HT adsorbs cationic dye. Physical adsorption in pore canals is secondary, and the discovered adsorption reaction occurs spontaneously by adsorption thermodynamic analysis.

## Figures and Tables

**Table 1:** Cross-reference between temperature and saturated vapor pressure.

Temperature (°C)	100	150	200	250
Saturated vapor pressure (×10^3^ Pa)	101.32	475.72	1553.60	3973.60

**Table 2:** Results of physical characteristics of adsorbents.

Adsorbent	BET Surface Area (m^2^/g)	Pore Volume (cm^3^/g)	Average Pore Size (nm)	Porosity (%)	Scatter Ratio (%)	Ignition Loss (%)
CM	11.14	0.0615	22.0837	63.04	0.74	28.55
CM-HT	18.64	0.1289	26.4947	69.54	2.58	29.39

**Table 3:** The root mean square error of results of physical characteristics of adsorbents.

		Sum of Squares	Degree of Freedom	Mean Square	F	Significance
BET surface area	With group	84.375	1	84.375	758.768	<0.01
	Inter group	0.445	4	0.111		
	Total	84.820	5			
Pore volume	With group	0.007	1	0.007	2496.602	<0.01
	Inter group	0.000	4	0.000		
	Total	0.007	5			
Average pore size	With group	63.440	1	63.440	56.131	0.02
	Inter group	4.521	4	1.130		
	Total	67.961	5			
Porosity	With group	29.185	1	29.185	516.191	<0.01
	Inter group	0.226	4	0.057		
	Total	29.412	5			
Scatter ratio	With group	5.078	1	5.078	330.840	<0.01
	Inter group	0.061	4	0.015		
	Total	5.140	5			
Ignition loss	With group	1.058	1	1.058	0.822	0.416
	Inter group	5.150	4	1.288		
	Total	6.209	5			

**Table 4:** Influence on *d*_001_ of clay minerals after hydrothermal modification.

Mineral	*d*_001_ Value/Å of Face Net	
	Before Modification	After Modification
Montmorillonite	12.6176	16.2916
Rectorite	24.3743	26.7583

**Table 5:** Nonlinear regression parameters of isotherm curves for MG adsorption of both adsorbents.

Absorbent	*q_e,exp_* (mg/g)	Langmuir Equation			Freundlich Equation		
		*q_max,fitted_* (mg/g)	*K_L_*·(L/mg)	R^2^	*N*	*K_F_* [mg·g^−1^·(mg·L^−1^)^−1/n^]	R^2^
CM	185.10	310.12	0.13217	0.9832	5.30	52.37	0.9841
CM-HT	290.45	625.15	0.20268	0.9938	5.89	118.68	0.9902

**Table 6:** Parameters of kinetic model fits for CM and CM-HT adsorbing MG.

Adsorbent	*q_e,exp_* (mg/g)	Pseudo-First Kinetic Model			Pseudo-Secondary Kinetic Model		
		*q_e_*_1_ (mg/g)	*k*_1_ (/min)	R^2^	*q_e_*_2_ (mg/g)	*k*_2_ (mg/g/min)	R^2^
CM	185.10	218.47	0.00408	0.9965	263.24	2.3 × 10^−5^	0.9943
CM-HT	290.45	321.17	0.00495	0.9732	331.10	6.4 × 10^−5^	0.9455

**Table 7:** Thermodynamic parameters for CM and CM-HT adsorbing MG dye.

Absorbent	Temperature (K)	Δ*G* (kJ/mol)	Δ*H* (kJ/mol)	Δ*S* (kJ/mol/K)	R^2^
CM	288	−33.432	−20.8906	0.0438	0.9548
	298	−34.076			
	308	−34.458			
	318	−34.767			
CM-HT	288	−29.978	−9.9373	0.0694	0.9969
	298	−30.632			
	308	−31.290			
	318	−32.075			

## Data Availability

The data presented in this study are available on request from the corresponding author. The data are not publicly available due to our following related articles are currently in the submission process.
